# Case report: Joint deformity associated with systemic lupus erythematosus

**DOI:** 10.1002/iid3.717

**Published:** 2022-09-28

**Authors:** Shu‐Lin Chen, Hui‐Juan Zheng, Li‐Yu Zhang, Qiang Xu, Chang‐Song Lin

**Affiliations:** ^1^ Department of Rheumatology The First Affiliated Hospital of Guangzhou University of Chinese Medicine Guangzhou China; ^2^ The First Clinical Medicine School Guangzhou University of Chinese Medicine Guangzhou China

**Keywords:** bone erosion, Jaccoud's arthropathy, rheumatoid arthritis, rhupus, systemic lupus erythematosus

## Abstract

**Objective:**

Typically, Jaccoud arthropathy (JA) is characterized by joint deformation without bone erosion. However, some recent studies have shown that bone erosion also occurs in JA; however, this remains controversial. To date, there have been no unified diagnostic standards for JA. Herein, we report a case of systemic lupus erythematosus complicated with JA without bone erosion.

**Methods:**

A 27‐year‐old woman was admitted to our department with a 2‐year history of pain, swelling, and progressive deformities of her hands and feet. She was diagnosed with systemic lupus erythematosus and class V lupus nephritis 5 years prior. Upon examination, her erythrocyte sedimentation rate and C‐reactive protein levels were found to be increased. She was positive for antinuclear antibodies, antidouble stranded DNA antibodies, and antiextractable nuclear antigen antibodies, with a decreased complement C3 and C4. Radiography and magnetic resonance imaging revealed no bone erosion. The patient was diagnosed with JA. She was treated with oral prednisone (10 mg daily), tofacitinib (5 mg twice daily), methotrexate (10 mg weekly), and celecoxib (0.2 g twice daily).

**Results:**

The patient's joint symptoms improved after treatment. No further progress was observed during the 4‐month follow‐up period.

**Conclusion:**

We believe that bone erosion is the key to distinguish rhupus syndrome from JA. However, this needs to be confirmed with further long‐term follow‐up studies. We found that the use tofacitinib, MTX, and celecoxib in combination with prednisone may be an effective regimen for the treatment of JA.

## Graphical abstract

We report a case of systemic lupus erythematosus (SLE) with Jaccoud's arthropathy (JA). We believe that SLE with erosive joint deformities should not be diagnosed as JA but rather as rhupus. We present this case to highlight the distinctions to consider during diagnosis, which would be of importance in clinical practice.

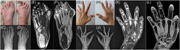

## 1 INTRODUCTION

In 1866, ES. Jaccoud first described Jaccoud's arthropathy (JA) as deforming a arthropathy in a patient with rheumatic fever and heart valve disease.^1^ Typically, JA is characterized by joint deformities of the hands and feet without bone erosion.^2^ The joints may be swollen or tender, with typical joint deformities, such as a swan neck, Z‐deformity of the thumb,^3^, ^4^ ulnar deviation, boutonniere deformity, and hallux valgus.^5^ Ligament and joint capsule relaxation are mechanisms of joint deformities.^6^ Gradual JA has been observed in dermatomyositis,^7^ psoriatic arthritis,^8^ systemic sclerosis,^9^ Sjögren's syndrome, and other diseases.^10^ It is the most common in systemic lupus erythematosus (SLE), with a prevalence of 1%–5%.^11^, ^12^

To date, there are no unified diagnostic standards for JA. Spronk et al. scored the number of affected fingers and the joint deformities, defining JA with a total score >5,^13^ while Van Vugt et al. proposed that deforming arthropathy associated with SLE without bone erosion should be divided into “JA” and “mild deforming arthropathy”,^14^ and Santiago further proposed that rheumatoid arthritis (RA) and other diseases with joint symptoms should be excluded before a JA diagnosis was made.^15^

With increasing research, some studies have shown that bone erosion also exists in JA^16^; therefore, it has become controversial whether JA is erosive. Herein, we report a case of SLE with JA. At her most recent admission to our center, the patient, diagnosed previously with SLE, was experiencing joint stiffness and pain, as well as had significant physical deformities in her hands and feet. Laboratory examination showed evidence of a heightened inflammatory response and positive autoimmune serology with an increased complement C3 and C4. Moreover, her radiography and magnetic resonance imaging (MRI) did not reveal bone erosion and the patient was diagnosed with SLE complicated by JA. In this and other cases, we believe that joint deformities with bone erosion should not be diagnosed as JA but rather as RA. We present this case to highlight the distinctions to consider during diagnosis.

## 2 CASE DESCRIPTION

This was a retrospective investigation of a case of JA in a patient admitted to the First Affiliated Hospital of Guangzhou University of Chinese Medicine. The work described was exempted by the Ethics Committee of The First Affiliated Hospital of Guangzhou University of Chinese Medicine because it did not involve identifiable personal information. The patient provided informed consent to this study. The study was conducted in accordance with the Declaration of Helsinki.

In January 2015, a 22‐year‐old woman was admitted to our hospital with a chief complaint of heart palpitations, weakness, and poor appetite over the previous month. Upon examination, it was determined that the patient had leucopenia, mild anemia, low complement levels, and elevated erythrocyte sedimentation rate (ESR) and C‐reactive protein (CRP) levels. The patient was positive for antinuclear antibodies (ANA), antidouble‐stranded DNA (dsDNA) antibodies, anti‐Smith antibodies, antihistone antibodies (AHA), and antinucleosome antibodies (AnuA). In addition, class V lupus nephritis was confirmed by renal biopsy. Therefore, she was diagnosed with SLE and in remission after immunomodulatory therapy with prednisone, methotrexate (MTX), hydroxychloroquine, and total glucosides of paeony (TGP).

In 2018, the patient developed swelling and pain in her right metatarsophalangeal (MTP) joint, accompanied by morning stiffness. The medication regimen was adjusted several times, but there was no apparent improvement in joint pain. Joint deformities were also observed. In 2019, she gradually developed bilateral multiple joint pain in the shoulders, elbows, wrists, interphalangeal joints, and knees in addition to continued morning stiffness and evident joint deformities. Therefore, her medication regimen was adjusted to prednisone (20 mg by mouth daily), tofacitinib (5 mg by mouth twice daily), and MTX (10 mg by mouth weekly). However, her symptoms did not improve, and she was admitted to in‐patient treatment at our rheumatology department.

**Figure 1:**
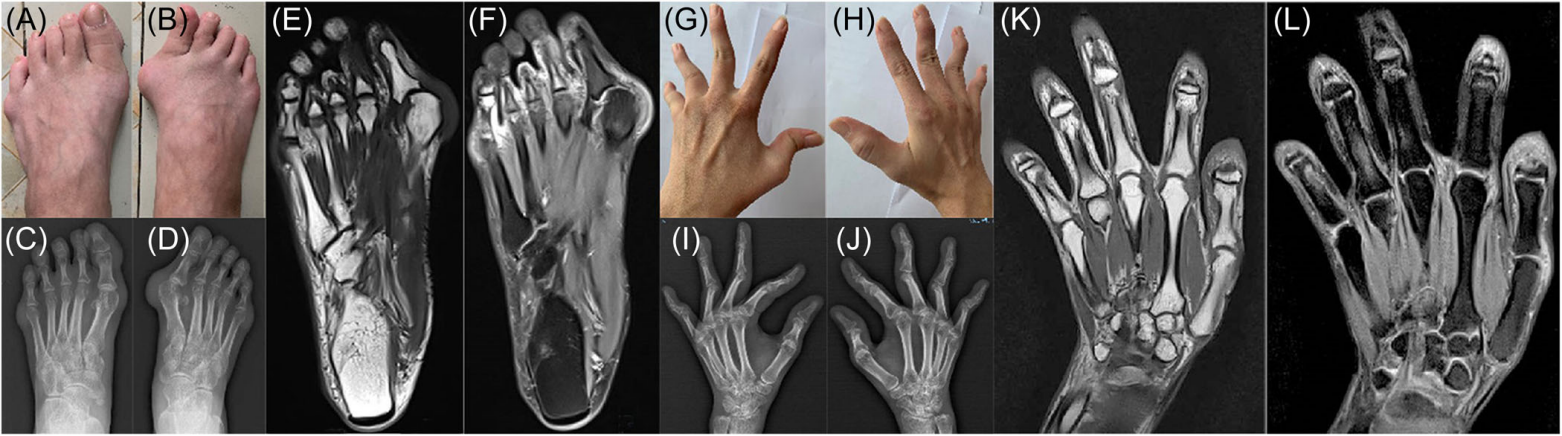
Jaccoud's arthropathy. (A, B) Clinical photographs of both feet with swollen joints and hallux valgus. (C, D) Radiography of both feet with luxation and subluxation of the joints. (E, F) The magnetic resonance image (MRI) of the right foot with synovitis. (G, H) Clinical photographs of both hands with swollen joints, swan neck deformities, and ulnar deviation. (I, J) Radiography of both hands with luxation and subluxation of the joints. (K, L): the MRI of the right hand with synovitis.

On admission, the patient had joint pain and shortness of breath. The shortness of breath occurred after activity, and no lesions were found on chest radiography and transthoracic echocardiography. This symptom may have been related to an impaired lung function caused by interstitial lung disease. However, the patient did not undergo a chest CT to confirm this for her limited economic capacity. She had no oral ulcers, photosensitivity, facial erythema, rash, hair loss, signs of Raynaud's phenomenon, rheumatoid nodules or symptoms and signs of other connective tissue diseases (CTDs), such as Sjogren's syndrome, scleroderma or psoriatic arthritis. Her medical history was unremarkable. She had no family history of premature bunion deformity, RA, SLE, psoriasis or any other CTDs. Physical examination revealed a bilateral hallux valgus, swan neck deformity involving the bilateral third to fifth digits, and an ulnar deviation at the bilateral metacarpophalangeal (MCP) and MTP joints (Figure 1A,B,G,H). The joint deformities were non‐reducible. A laboratory examination showed increased ESR (24 mm/h) (reference range [RR] 0–20 mm/h), d‐dimer (3.1 mg/L) (RR 0–0.55 mg/h), and CRP levels (27.5 mg/L) (RR 0–8 mg/L). Autoimmune serology was positive for ANA (1:3200, homogeneous pattern), anti‐dsDNA antibodies, AHA, and AnuA, along with an increase in complement C3 (0.609 g/L) (RR 0.79–1.52 g/L) and C4 (0.141 g/L) (RR 0.16–0.38 g/L). Rheumatoid factor (RF) and anticitrullinated protein antibodies (ACPA) tests were negative. Radiography of the hands and wrists showed luxation and subluxation of the MCP and interphalangeal joints (Figure 1I,J). The same features were observed in the patient's feet (Figure 1C,D). MRI revealed that the joint surface of the right foot had cystic degeneration with subcutaneous soft tissue swelling (Figure 1E,F), and the left hand had soft tissue swelling around the joint (Figure 1K,L). However, no evidence of erosion was found on the articular surfaces.

**Figure 2:**
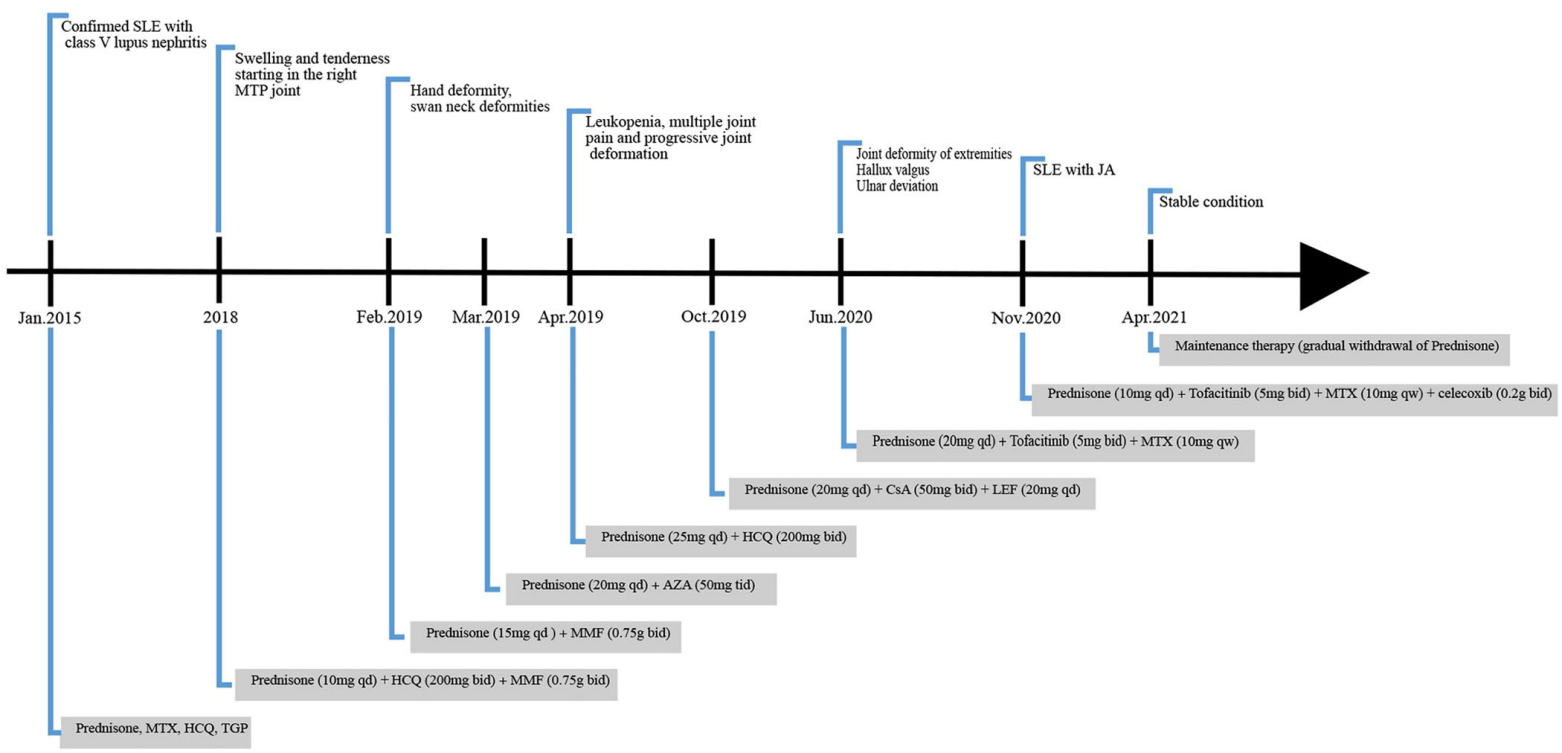
The timeline of clinical manifestations and treatments. All medicines were taken orally. AZA, azathioprine; bid, twice daily; CsA, cyclosporine A; HCQ, hydroxychloroquine; LEF, leflunomide; MMF, mycophenolate mofetil; MTX, methotrexate; qd, daily; qw, weekly; TGP, total glucosides of paeony; tid, three times daily.

The patient was diagnosed with SLE complicated by JA. The medication regimen was adjusted to prednisone (10 mg by mouth daily), tofacitinib (5 mg by mouth twice daily), MTX (10 mg by mouth weekly), and celecoxib (0.2 g by mouth twice daily); the patient showed steady improvement and was discharged from the hospital. Regular follow‐up visits were conducted at the outpatient clinic. During maintenance therapy for the gradual withdrawal of prednisone, the patient's joint pain was controlled, and no further disease progression was observed. The follow‐up laboratory results indicated improvement in the d‐dimer (1.64 mg/L) level. Details of the clinical manifestations and treatment processes are shown in Figure 2.

## 3 DISCUSSION

JA is an arthropathy characterized by joint deformation without bone erosion. The patient described herein had a history of SLE accompanied by deformities of the hands and feet. The patient's radiography showed joint dislocation but no bone erosion, which is consistent with the diagnosis of JA. However, it is controversial whether JA is erosive.

Our report confirmed that although bone erosion can be detected more accurately using ultrasound and MRI than with radiography, there are still some patients with JA that show no bone erosion. In addition to our report, there have been several studies describing JA patients without bone erosion based on MRI findings.^16^, ^17^, ^18^, ^19^ This may indicate that joint deformities with or without bone erosion are two different types of diseases.

In a study by Santiago et al., where ultrasound and MRI scans were used to examine the joints of seven SLE patients with severe JA, bone erosion was observed in only one patient.^18^ Shi et al. reported a case of SLE with progressive deformities of the hands and feet.^20^ The radiography of this patient showed erosive bone lesions, and the patient was diagnosed with erosive JA. Both patients were negative for ACPA and RF; however, the synovitis, ESR, and CRP status of the patients were not mentioned, and therefore, RA diagnosis cannot be excluded. Similar flaws appeared in the findings of Ribeiro et al., Lins et al., and Sá Ribeiro et al.^16^, ^21^, ^22^

While there are currently no diagnostic criteria for RA, classification criteria can be used to assist the diagnosis.^23^ When a JA patient has more than 10 joints involved, accompanied by synovitis and elevated CRP levels or ESR, RA can be diagnosed according to the 2010 American College of Rheumatology (ACR)/European Alliance of Association for Rheumatology RA classification criteria.^24^ When a JA patient with symmetrical arthritis has at least three joint areas including the hands involved, and the radiography scan shows bone erosion, RA can be diagnosed according to the 1987 ACR criteria.^25^ Based on this, a case of a female JA patient with rheumatic fever and joint deformities of the hands reported by Beausang et al. and another patient with SLE and JA reported by Saketkoo and Quinet fulfilled the diagnostic criteria for RA.^26^, ^27^ While it may not be appropriate to use the RA classification criteria to diagnose arthritis in patients with rheumatic fever or SLE, using the RA classification criteria as a reference, “JA with bone erosion” indicates some of the characteristics of RA. Although this arthritis may not be RA, it should be distinguished from “JA without bone erosion” because the effects of bone erosion on joints are different from those without bone erosion. It is worth noting that reports of JA with bone erosion do not appear in association with scleroderma or psoriasis; however, a focus on SLE, may indicate that the manifestation of bone erosion is related to the primary disease.^28^, ^29^ SLE patients have bone erosions that are often underestimated, and the mechanism may be related to chronic inflammation.^30^, ^31^ Although some SLE patients may have joint symptoms, there are still some symptoms that cannot be explained by SLE. Considering the differences between the clinical manifestations and the disease outcome, as well as the impact on the quality of life (simple joint pain or inability to twist the towel with deformed fingers), it is necessary to distinguish deforming arthropathy complicated with SLE from simple joint symptoms. Ostendorf et al. proposed that patients with SLE and erosive arthritis should be diagnosed with rhupus, an independent disease characterized by the combination of RA and SLE.^17^ The diagnosis of rhupus fulfills the diagnostic criteria for both SLE and RA. Some of the established criteria also include bone erosion, positive RF, and ACPA, while others do not.^32^ Due to the unclear criteria, JA and rhupus may be confused. However, the 1987 ACR criteria included bone erosion in the RA diagnostic criteria, and inflammatory bone erosion is a characteristic manifestation of RA.^33^ Rhupus is characterized by bone erosion, similar to RA.^34^ In contrast, JA is characterized by joint deformation, without bone erosion. Therefore, we believe that the presence of bone erosion should be regarded as the key to distinguishing rhupus from JA. Since erosive JA also shows characteristics of RA, we proposed that patients with SLE and erosive JA should be regarded as having rhupus. Contrary to the traditional view, some literatures have reported that JA patients without radiography findings can be detected fewer and smaller bone erosion than rhupus patients through ultrasound, CT and MRI.^35^, ^36^ Therefore, the severity of bone erosion may be the real key to distinguish the two diseases. However, it is not excluded that these SLE patients may actually have rhupus. Biopsy was not performed due to patient refusal. It is thought that the characteristic pathology of JA is tendon and ligament changes. However, this may also occur in a variety of inflammatory arthritis, including RA.^37^ Synovitis is also not specific too. In contrast, bone erosion is more specific for the identification of arthritis.

Due to the unclear pathogenesis, there is no specific treatment for JA complicated with SLE. Anti‐inflammatory drugs, corticosteroids, MTX, and antimalarials are used commonly.^6^ In our study, TGP (0.6 g by mouth twice daily) were used in the early treatment. TGP is the extract of the dried root of *Paeonia lactiflora* Pallas, it has anti‐inflammatory and immunomodulatory effects and is used to treat a variety of autoimmune diseases, including SLE and RA.^38^ Our study was the first case to report the use of Janus kinases inhibitors in the treatment of JA. The patient used mycophenolate mofetil, azathioprine, hydroxychloroquine, cyclosporine A, leflunomide successively, and the joint symptoms continued to progress. After using tofacitinib, MTX, and celecoxib in combination with prednisone, the progression of joint symptoms in the patient slowed down, and the joint pain and edema were reduced. We observed that the patient's d‐dimer levels declined after the use of the combination therapy. d‐dimer is a biomarker of immune inflammation.^39^ Studies have shown that tofacitinib can be used to treat SLE and its complicated premature atherosclerosis.^40^, ^41^ The development of JA is associated with longer duration and lack of immunosuppressive therapy, and JA‐related synovitis suggests a potential risk of SLE disease activity, which may indicate the therapeutic potential of tofacitinib in JA.^42^ In this study, we found that the use of tofacitinib, MTX and celecoxib in combination with prednisone can also control the progression of JA complicated with SLE. These three drugs have been shown to be effective in the treatment of RA, which may indicate the potential efficacy of the RA combination regimen applied to JA.^43^ Drugs of RA may be more widely used in the treatment of JA, and biologic disease‐modifying antirheumatic drugs such as abatacept or tocilizumab may be potentially effective. Further research is needed to confirm this hypothesis.

In conclusion, JA is a joint disease associated typically with deformation but not bone erosion. However, some studies have shown that JA is bone erosive. We believe that patients with SLE and erosive JA should be regarded as having rhupus and we posit that bone erosion is the key to distinguishing rhupus from JA. Moreover, we found that the use tofacitinib, MTX, and celecoxib in combination with prednisone may be an effective regimen for the treatment of JA. This conclusion needs to be verified through long‐term follow‐up studies. Clinically, joint pain in SLE patients tends to be regarded as an accompanying symptom. The patient's joint symptoms may be overlooked in the treatment of the primary disease. However, when the “joint pain” is actually JA or rhupus, this may lead to impaired joint function. We recommend the regular examination of the joints of SLE patients with joint symptoms. We recommend an increased focus on SLE complicated with erosive and non‐erosive arthritis.

## AUTHOR CONTRIBUTIONS

Shu‐Lin Chen, Hui‐Juan Zheng, and Li‐Yu Zhang collected the data and drafted the manuscript. Qiang Xu conceived of the study and participated in designing and writing this manuscript. Chang‐Song Lin reviewed and revised this manuscript. All authors contributed to the article and approved the submitted version.

## CONFLICT OF INTEREST

The authors declare no conflict of interest.
